# Editorial: Developing sprinters: how can we swim, cycle, and run faster?

**DOI:** 10.3389/fspor.2025.1734106

**Published:** 2025-11-18

**Authors:** Jesús J. Ruiz-Navarro, Daniel Boullosa, Marina Gil-Calvo, Dennis-Peter Born

**Affiliations:** 1 Aquatics Lab, Department of Physical Education and Sports, Faculty of Sport Sciences, University of Granada, Granada, Spain; 2 Faculty of Physical Activity and Sports Sciences, Universidad de León, León, Spain; 3 Integrated Institute of Health, Federal University of Mato Grosso do Sul, Campo Grande, Brazil; 4 College of Healthcare Sciences, James Cook University, Townsville, QLD, Australia; 5 Swiss Development Hub for Strength and Conditioning in Swimming, Swiss Aquatics—National Swimming Federation, Worblaufen, Switzerland; 6 Department for Elite Sport, Swiss Federal Institute of Sport Magglingen, Magglingen, Switzerland

**Keywords:** swimming, track and field, athletics, biomechanics, physiology, performance

Swimming, cycling, and track and field are among the most-watched sports in the Olympic Games. Despite the complex interaction between aerobic and anaerobic energy supply, sprint performances in these three sports heavily rely on rapid energy production from anaerobic metabolism. Although event durations and intensities may be similar between sports, the training regimens of swimmers, cyclists, and track and field athletes differ considerably and provide the opportunity for cross-disciplinary learning. Therefore, this research topic aimed to gather insights into the determinants of sprint performances across these sports to develop faster athletes.

Because water does not provide sufficient resistance for substantial strength gains from sport-specific movements, swimmers typically perform several dry-land strength training sessions per week to provide an overload stimulus to the neuro-muscular system (1). In this regard, Venâncio et al. explored the evolution of research on strength training in swimmers. Their review reveals an exponential increase in research interest, particularly since 2010. Moreover, strength training has been predominantly related to sprint events in the swimming literature. This growing body of evidence encompasses a wide range of countries, highlighting the importance of incorporating strength training into swimmers’ programs while respecting each athlete's unique characteristics and the specific demands of their events.

Resisted and assisted sprints are often used to enhance speed and acceleration capabilities (2). Following advancements in motorized resistance devices (MRD), Eriksrud and Westheim assessed intrasession and test-retest reliability of assisted running sprint outcomes. They reported high to extremely high intra-class correlations and, generally, good coefficients of variation for both test–retest and intrasession reliability, hence supporting the use of MRD for training and evaluation. In addition, Jiménez-Reyes et al. investigated the impact of varying overload conditions on the mechanical determinants of runners’ performance during sprint acceleration. Their results indicated that heavy loads mainly affect the early acceleration phase, while light loads provide a broader range of mechanical stimulation. These findings can guide load selection, thereby improving effectiveness and specificity of resistance- and assistance-based sprint training.

MRD has also facilitated the assessment of in-water swimmers’ physical capabilities through the load–velocity (LV) profile (3). Sengoku et al. examined the usefulness of LV profiling by investigating the relationship between maximal lactate accumulation rate (ċLa_max_) and sprint performance parameters. The ċLa_max_ was associated with the theoretical maximal load that a swimmer can pull during front crawl swimming and 50 m front-crawl performance, particularly during the initial meters of the event. These results highlight the contribution of higher glycolytic power to faster performance at the start of a sprint race. As most studies have used cross-sectional designs (4, 5), Keating et al. investigated LV profiling and competition performance in national- and international-level swimmers over 15 months. Although fluctuations were observed across the evaluated period, no differences emerged relative to the baseline measurements. However, differences were observed between performance levels, with international-level swimmers showing greater stability than national-level swimmers. These findings support LV profiling as a valuable tool for swimmers’ monitoring over time, contributing to more effective training prescription.

The inflammatory response to exercise, which is influenced by myokine release and modulated by fat-free mass, biological maturation, and dietary inflammatory index, may be considered when manipulating training loads (6). Almeida-Neto et al. investigated the effect of these factors on myokine release following repeated sprint training. The results showed that athletes with higher fat-free mass or greater maturity evidenced smaller inflammatory variations, hence showing potential for faster recovery between high-intensity sessions. Moreover, diets with lower inflammatory potential were linked to more efficient immune responses, thus highlighting the importance of nutrition to reduce exercise-induced immune stress, improve recovery, and enhance performance.

Given the comparable competition formats in swimming and track running competitions (7, 8), Born et al. compared performance progression and variety in race distances of comparable lengths (timewise) between swimmers and runners. Sprint swimmers exhibited a wider variety of race distances than runners. However, distance variety was not a fixed continuum, but rather an evolving process throughout the female athletes’ careers (Born et al.). While swimmers generally exhibited greater variety than track runners, progressive specialization with advancing age increased the likelihood of achieving international-class swimming performance. Taken together, both studies suggest that sprint swimmers may benefit from earlier and more pronounced specialization to maximize their physiological potential.

Additionally, stroke specialization appears to be an important performance contributor to the technical adjustments. As such (9), Yamakawa et al. examined the effects of different breathing patterns on muscle activity and coordination in butterfly sprint swimming. Although breathing every compared to every second stroke did not affect swimming speed, it altered muscle synergies, leading to an earlier onset of muscle activity. These findings highlight the need for race pace-specific training, implementation of the specific breathing patterns, and neuro-muscular preparation during dry-land training for the improvement of specific muscle synergies.

As pacing is another key factor for sprint performance (10), Yoshimoto et al. investigated the relationship between 200 m running performance and pace distribution. World-class athletes typically run relatively slower in the first half. Similarly, a within-subject analysis showed that a faster second-half speed was linked to better overall performance time. As such, moderating early acceleration and maintaining speed later in the race may enhance the overall race outcome in 200 m sprint running.

In conclusion, this special issue offers valuable, state-of-the-art insights into the development of human sprint performances. Through comparisons across multiple sports, it presents evidence that can inform and enhance coaching and training methodologies. Specifically, the findings highlight the importance of strength training and provide guidance for optimal load adjustments of resisted sprinting, reliability of assisted sprints, and the use of MRD for longitudinal assessment of physical capacities. The findings also show the individual response to repeated sprints, underscoring the need for personalized programming in sprint training. As swimmers may benefit from greater specialization than track runners, sprint training should be designed to target the specific technical and pacing demands of the competition. Finally, we would like to thank the authors and reviewers for their support and efforts with this research topic.
